# Micro-concave waveguide antenna for high photon extraction from nitrogen vacancy centers in nanodiamond

**DOI:** 10.1038/srep12013

**Published:** 2015-07-14

**Authors:** Ranjith Rajasekharan, Günter Kewes, Amir Djalalian-Assl, Kumaravelu Ganesan, Snjezana Tomljenovic-Hanic, Jeffrey C. McCallum, Ann Roberts, Oliver Benson, Steven Prawer

**Affiliations:** 1 Department of Electrical and Electronic Engineering, The University of Melbourne, Melbourne, Victoria 3010 Australia; 2 Nano-Optics, Institute of Physics, Humboldt-Universität zu Berlin, Newtonstraße 15, D-12489 Berlin, Germany; 3 School of Physics, The University of Melbourne, Victoria 3010, Australia

## Abstract

The negatively charged nitrogen-vacancy colour center (NV^−^ center) in nanodiamond is an excellent single photon source due to its stable photon generation in ambient conditions, optically addressable nuclear spin state, high quantum yield and its availability in nanometer sized crystals. In order to make practical devices using nanodiamond, highly efficient and directional emission of single photons in well-defined modes, either collimated into free space or waveguides are essential. This is a Herculean task as the photoluminescence of the NV centers is associated with two orthogonal dipoles arranged in a plane perpendicular to the NV defect symmetry axis. Here, we report on a micro-concave waveguide antenna design, which can effectively direct single photons from any emitter into either free space or into waveguides in a narrow cone angle with more than 80% collection efficiency irrespective of the dipole orientation. The device also enhances the spontaneous emission rate which further increases the number of photons available for collection. The waveguide antenna has potential applications in quantum cryptography, quantum computation, spectroscopy and metrology.

Compared to strategies developed to increase coupling and collection efficiency from single photon sources such as single molecules, quantum dots and most single defect centres^1^,^2^,^3^,^4^,^5^,^6^,^7^,^8^,^9^; nitrogen vacancy colour centers (NV centers )^10^,^11^,^12^ in both bulk and nanodiamond still represent great challenges^13^,^14^,^15^,^16^,^17^,^18^. The main difficulties associated with the NV centre in a diamond are i) a broad emission band ii) emission in a high index medium (*n* = 2.4) and iii) emission associated with two orthogonal dipoles arranged in a plane perpendicular to the NV defect symmetry axis^10^. The absolute orientation of the two orthogonal dipoles is determined by the direction of nonaxial local strain in a diamond matrix^10^ which is randomly distributed in the diamond matrix. Many strategies have been developed to increase the collection efficiency and control directionality from the NV centres and/or to enhance the emission rate through the Purcell effect such as using solid immersion lenses^19^,^20^, photonic crystals^13^,^19^,^21^,^22^, fibre tips^14^, waveguides^23^,^24^,^25^,^26^,^27^,^28^, microspheres, microcavities^9^,^10^,^11^,^12^,^13^,^14^,^15^,^16^,^17^,^18^,^19^,^20^,^21^,^22^,^23^,^24^,^25^,^26^,^27^,^28^,^29^,^30^,^31^,^32^ and the use of diamond nanowires^33^,^34^.

The drawbacks of many of these approaches are that such schemes developed for enhancing the collection efficiency are sensitive to the dipole orientation of the emitter and the accuracy of positioning the emitter relative to an optical structure. In systems with very small mode volumes, for example in plasmonics^3^,^4^,^35^ or photonic crystals, the accurate positioning of the dipole can be very difficult. In plasmonic systems, nonradiative loss channels can open up when the dipole emitter is close to the metal. Furthermore, the design can often be optimised for only parallel or perpendicular dipole orientations. Since the NV centre has two orthogonal dipoles, it is extremely difficult to control the orientation during integration into a nanophotonic device, particularly when nanodiamond is used. Thus, to establish an antenna for the NV centres with high effective photon output and technical feasibility, we aim for a design that is relatively insensitive to emitter dipole orientation and less sensitive to positioning of the emitter. Also the device geometry should be sufficiently broadband to collect the whole phonon side-band. Most importantly the aim is to direct the emission into a sufficiently narrow angle to achieve high photon rates even with dry objective (oil immersion objectives prevent developing portable devices) of poor numerical apertures/ fibers and also when long working distances are inevitable.

Here, we report on a theoretical study of a novel feasible micro-cavity waveguide antenna design, which can effectively direct photons from a single NV center in a nanodiamond into free space or a waveguides in a very narrow cone angle with more than 80% collection efficiency irrespective of the dipole orientations. The proposed antenna enhances the spontaneous emission rate which further increases the number of photons available for collection. It is also insensitive to the location of the dipole in the antenna over a range of distances which are well within any fabrication tolerances.

## Results

### Evolution of cavity waveguide antenna from a planar waveguide

**Figure 1:**
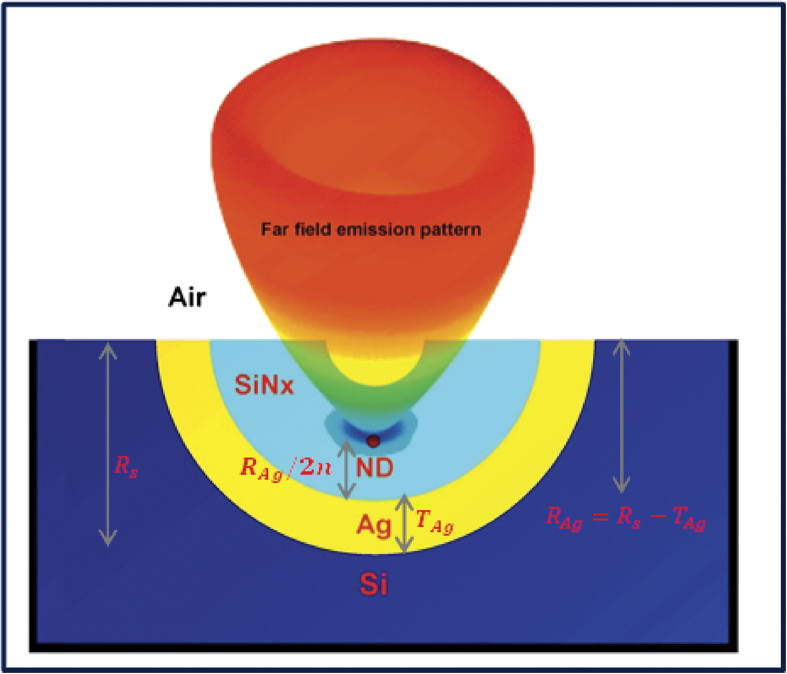
Schematic diagram of the mirco-cavity waveguide antenna and far field emission intensity from a nanodiamond (ND) which is placed at the centre of the antenna buried in silicon nitride layer with a height (t ~ *R*_*Ag*_/2*n*) from the silver surface.

Figure 1 shows a schematic diagram of the cavity waveguide antenna together with the far field emission pattern from a nanodiamond (ND) placed in the antenna. The geometry consists of a semispherical concave surface on a silicon substrate with radius *R*_*s*_ covered with a silver film of thickness *T*_*Ag*_ to form a cavity with radius of curvature, *R*_*Ag*_(*R*_*Ag*_ = *R*_*s*_  –  *T*_*Ag*_). The thickness is chosen to be much greater than the skin depth of silver to act as a mirror. The silver is then covered with first layer of silicon nitride (refractive index 2) of thickness *T*_*SiNx*_ which is approximately equal to *R*_*Ag*_/2. Silicon nitride is chosen because of its low loss in the visible region and refractive index close to diamond (refractive index 2.4) to reduce light scattering due to the refractive index mismatch in addition to diamond’s compatibility with silicon nitride from a fabrication point of view^36^. A spherical nanodiamond with a radius of 40 nm and the dipole in its center is placed at the centre of the cavity on this layer of silicon nitride with a height *R*_*Ag*_/2n from the silver surface. This first silicon nitride layer of thickness (*T*_*SiNx*_ ≥ *D*_*e*_)sets the height of the emitter from the bottom of the concave mirror.

*D*_*e*_ is the geometric distance from top surface of concave mirror to the location of the NV center in the nanodiamond sphere. This dielectric thickness is important to act as a mirror for the dipole and also to prevent any nonradiative decay channels of the emitter with the metallic mirror. In practical applications, the nanodiamond can be placed at any specified location using an atomic force microscope (AFM) tip or tungsten probe attached in a SEM^37^,^38^. Then a second layer of silicon nitride of thickness *T*'_*SiNx*_ is deposited on the top of the emitter to form a waveguide in silicon nitride of thickness *T*_*SiNx*_ + *T*'_*SiNx*_.

**Figure 2:**
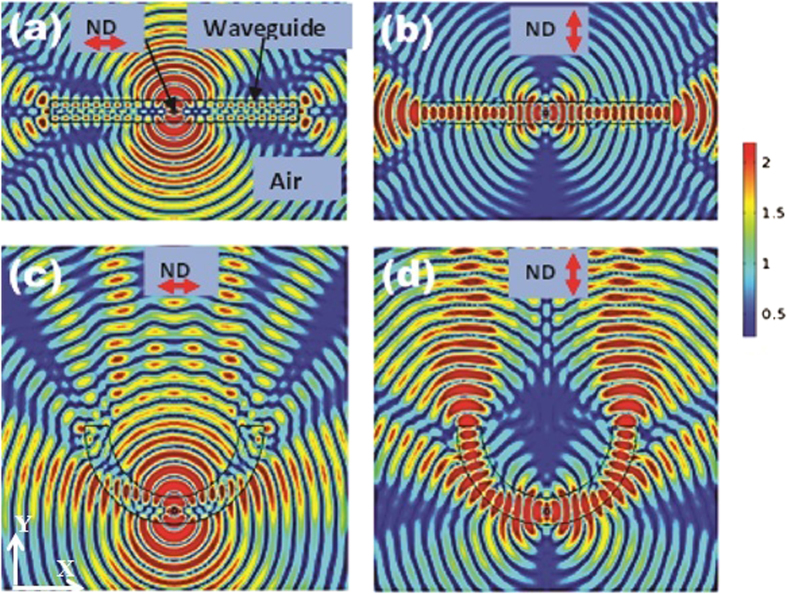
A dipolar emitter encapsulated in a sphere of radius 40 nm and the refractive index of 2.4 with parallel and perpendicular orientation placed inside two different waveguides (**a**) The dipole emitter placed in a planar waveguide of refractive index 1.5. The dipole orientation is along the *x* axis (parallel orientation) and emitted radiation is mostly directed along the ±*y* axis (**b**) when the dipole orientation is along y axis (perpendicular orientation), most of the emitted radiation is guided through the waveguide into both sides ±*x* (into four sides in 3D) (**c**) The emitter with parallel orientation, when the planar waveguide is transformed into a curved waveguide. The emitted radiation is mostly directed in the ±*y* axis (**d**) The emitter with perpendicular dipole orientation in the curved waveguide directs most of the emitted radiation into one direction (guided modes in the +*y* axis). The simulation area is 3.6 μm × 4.0 μm.

We start our study by showing how the emission from a single emitter with different dipole orientations behaves in a planar slab waveguide of thickness (Tw=300nm), length (Lw = 3 µm) and a refractive index of 1.5. This is followed by a gradual evolution of the planar slab into our novel micro-cavity waveguide antenna in three steps. The emitter is designed as a dipole, which emits at 700 nm vacuum wavelength embedded in a sphere of 40 nm radius and with refractive index 2.4 (~spectral peak of the NV- luminescence in nano-diamonds at room temperature). The detailed computational aspects are given in the Methods. Only two cases have to be considered viz, a parallel and a perpendicular dipole due to the symmetry of the problem. Figure 2(a) shows the emitted radiation (electric intensity) when the dipole orientation is parallel to the surface planar waveguide. Less than approximately half of the radiation is emitted through the top and bottom surfaces of the waveguide. A small fraction of the radiation meeting the criterion that the incident angle is above the critical angle at the waveguide-air interface is guided along the waveguide. Figure 2(b) shows the emitted radiation intensity when the dipole is perpendicular to the waveguide surface, where most of the radiation is guided with in the waveguide. Here, only a small fraction of emitted radiation can be collected even with a high numerical aperture (NA ~ 1.2 oil immersion) objective from one side of the planar waveguide.

As a first step, we transform the planar waveguide into a curved waveguide (here with curvature 
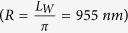
) in order to collect most of the emission from one side as shown in Fig. 2(c,d). For parallel dipole orientation, the curvature does not help to increase the collection efficiency as shown in Fig. 2(c) and hence only a fraction of the emitted radiation is collected from one side. However, for perpendicular dipole orientation as shown in Fig. 2(d), most of the emitted radiation is directed into one side (the radiation is directed into one side from two ends of the curved waveguide). Here, using a single high NA microscopic objective, most of the emitted photons can be collected for perpendicular dipole orientation in contrast to the planar waveguide. But, radiation still leaks through sides and the curvature does not work well for the parallel dipole orientation as shown in the Fig. 2(c).

**Figure 3:**
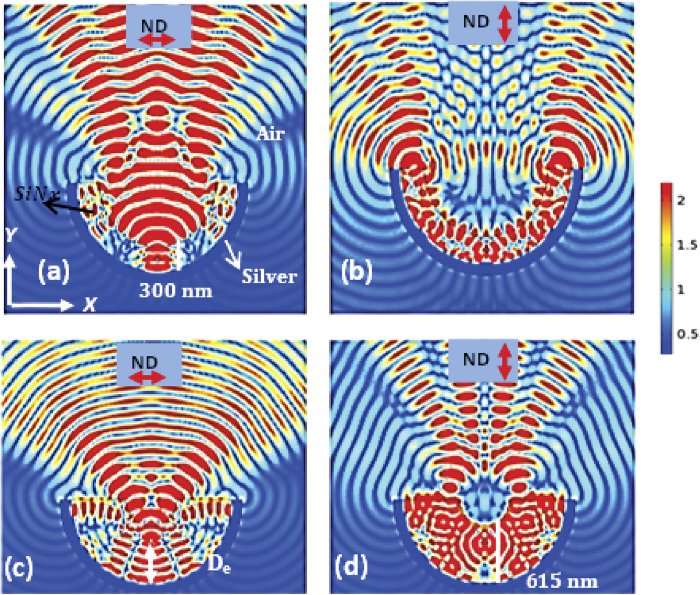
Evolution of a planar waveguide into a micro-cavity waveguide antenna (**a**) One side of the curved waveguide is coated with a thick silver film (300 nm) to reflect back all radiation leaking around the curved waveguide in the +*y* direction. The figure (**a**) shows emission from an emitter with ǁ orientation. The silver coated curved waveguide directs more than 98% emission into the upper half space (**b**) The emitter (_┴_) in the cavity directs both guided mode and leaking modes in one direction (**c**) The thickness of waveguide increased from 300 to 600 nm and the silver coated curved waveguide is transformed into a micro-concave waveguide antenna configuration. The emitter (ǁ) is placed at focal point (*f *= *R*/2n) of the micro-concave mirror with radius of curvature *R* (900 nm) to collimate into free space (**d**) The emitter (_┴_) placed at *f* + 50 *nm* (*De* > *f*).

To eliminate the above issues, in the second step a 300 nm thick silver is coated on the outer side of the curved waveguide as shown in Fig. 3. The silver coating acts as a mirror and the leaked radiation is reflected back. Fig. 3(a,b) show the effect of silver coating for parallel and perpendicular orientations of the emitter respectively. The new geometry guides all the emitted radiation into one direction (into a hemisphere) irrespective of the dipole orientation. However, using the new geometry, the emitted radiation is still not directed into a narrow cone angle to be collected using a waveguide or low NA objective lens or a fiber.

In the third step, we introduce a simple but powerful strategy by evolving this waveguide into a micro-cavity waveguide antenna configuration as shown in Fig. 3(c,d). In addition to the silver deposited on the outer side of the semicircular waveguide with radius of curvature *R*, the width of the waveguide is increased from 300 nm to 600 nm as shown in Fig. 3(c). The geometry now behaves like a concave mirror in addition to being a waveguide. A parabolic cavity design is preferred to spherical one to minimize chromatic and spherical aberration. A concave geometry is preferred over a parabolic one because of its relatively easy fabrication. Also a parabolic mirror is more sensitive to the position of the dipole due to its focal point. Using the micro-concave waveguide antenna, the emission from any emitter can be collimated or directed into a narrow cone angle with increased spontaneous emission rate irrespective of the dipole orientation (this will be quantified later) and with little dependence on location of the dipole in the waveguide antenna. For a concave mirror, the focal length is defined as 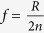, where *R* is radius of curvature of the cavity and *n* the refractive index of SiNx.

If the emitter is placed at the focal length (*D*_*e*_ = *f*), the light directed towards the mirror is collimated. But he light directed into half sphere not containing the mirror is diverging. Hence the total radiation coming out of the mirror is sum of these two contributions. When (*D*_*e*_ > *f*), the emitted light is directed into a cone angle. Figure 3(c) shows that the emitted radiation is collimated when the dipole (parallel) is placed at the focal point. Figure 3(d) shows the emitted light is directed into a narrow cone angle when the dipole is placed at f + 50 nm (*D*_*e*_ > *f*)for perpendicular dipole orientation.

### Micro-cavity waveguide antenna: a detailed study

**Figure 4:**
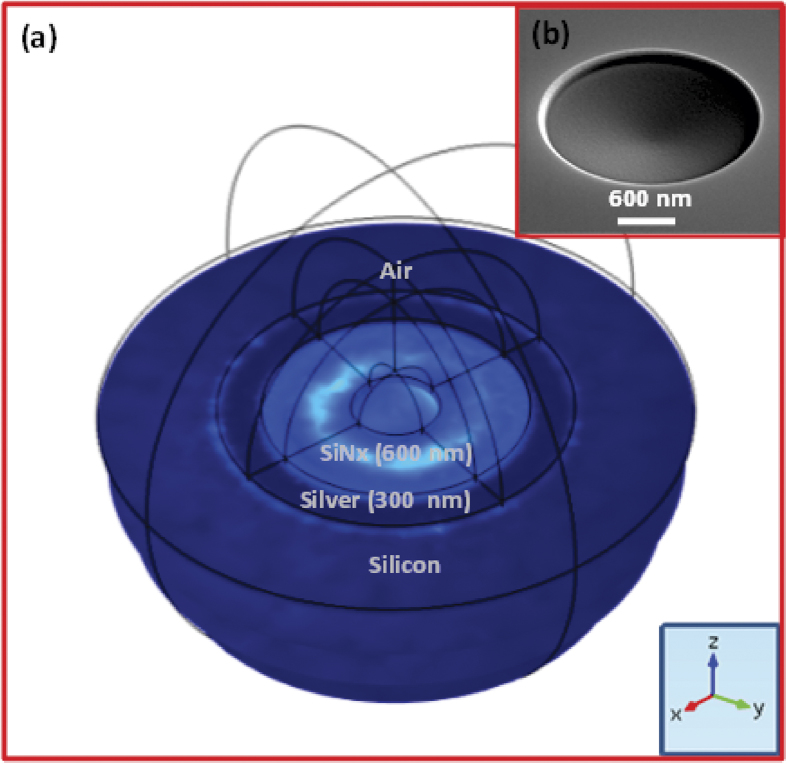
Computational geometry of the proposed micro-cavity waveguide antenna (**a**) 3D geometry of the antenna. The lower hemisphere consists of silicon substrate with a concave cavity covered with 300 nm silver (radius of curvature 900 nm) and 600 nm SiNx respectively. The full upper hemisphere is made of air. This geometry was used to calculate the far field emission pattern in 2D, 3D, emission cone angle and emission enhancement (**b**) SEM image of a cavity antenna of same dimension fabricated on silicon substrate.

**Figure 5:**
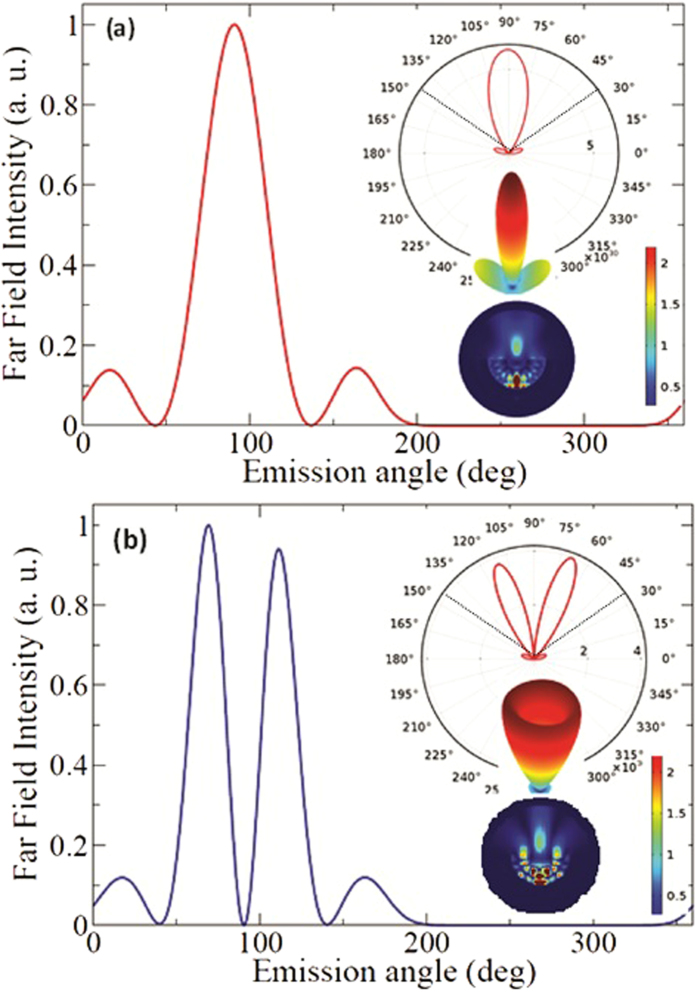
Far field radiation intensity pattern for ǁ and _┴_ dipole orientations in the micro- cavity waveguide antenna (**a**) Far field intensity with respect to emission angle for ǁ dipole orientation. The inset images show polar plot (2D), 3D far field intensity distribution and near filed intensity distribution for ǁ dipole. The dipole position is 315 nm from bottom of the cavity. The dotted line in the 2D polar plot corresponds to NA of 0.8 (**b**) Far field intensity vs emission angle for _┴_ dipole orientation along with polar plot, 3D far field intensity and near filed intensity distribution. The dipole position is 245 nm from the bottom of the cavity. The dotted line in the 2D polar plot corresponds to NA of 0.8.

Having explained the principle of the micro-cavity waveguide antenna, we present a detailed computational study of the antenna. The cavity antenna is computationally investigated in 3D using the finite element method (FEM) (details are given in Methods). The simulation geometry is shown in Fig. 4. The first parameter varied in a scan is the radius of curvature of the cavity, *R*_*Ag*_ from 200 nm to 1300 nm to find a suitable curvature for optimizing the radiation pattern for a fixed narrow cone angle. When the curvature is below 800 nm, the emitted radiation diverged at a large cone angle due to diffraction. Based on the collection efficiency, focusing power and minimum cavity radius, *R*_*Ag*_ is selected to be 900 nm. The parameters of the optimized micro-cavity waveguide antenna are *R*_*S*_ = 1200 *nm*, *T*_*Ag*_ = 300 *nm*, *R*_*Ag*_ = 900 *nm*, T_*SiNx*_ = 365 *nm for* ‖ and 315 for _┴_ dipole, and *T*′_*SiNx*_ = 300 *nm*. Using these optimized parameters, the far field radiation pattern for the parallel and perpendicular dipole orientations are calculated. We have shown in the inset of Fig. 4 that such a cavity is feasible and easy to fabricate on silicon (The inset shows SEM image of a cavity fabricated using focused ion beam). To quantify the insights gained from the simulations, we obtain the 3D far field emission profile. Figure 5 shows the far field intensity with respect to angle of emission, and 3D far field profiles for parallel (Fig. 5(a)) and perpendicular (Fig. 5(b)) dipole orientations, respectively. The plots show that more than 80% of the emitted power falls in between 50° and 140° (i.e. within a 90° full cone angle) for both of the dipole orientations. The corresponding numerical aperture (NA) is calculated as 0.707 using the equation NA = *nsinθ*, where *n* is the index of refraction of the medium in which the objective is working (for air, *n* = 1) and *θ* is the half-angle of the maximum cone of light that exit the antenna. The dotted line in the polar plots (the Fig. 5(a,b)) shows cone angle corresponding to a NA of 0.8 in air.

The emission intensity pattern is well within this cone angle. We have calculated the collection efficiency for different NAs starting from 0.95 to 0.4 for both ǁ and _┴_ as given in Table 1. For this calculation a fixed cavity with parameters same as Fig. 5 is used. The NA then varied from 0.95 to 0.4. The results show that the emission can be collected using an ordinary dry objective or a standard optical fiber. By adjusting the nanodiamond position *D*_*e*_ greater or smaller than the focal length of the cavity (*f*), it is possible to fine tune the emission (both the radiation pattern and total power emitted) depending on refractive index of the dielectric used for making the antenna.

The collection efficiencies calculated for parallel, perpendicular and 45 degrees dipole orientations are 85%, 81%, *and* 82% respectively (again referring to a 90° full cone angle). Furthermore we take a look at the enhancement of the spontaneous emission rate (SPE) of the nanodiamond in the antenna. The total emitted power from the dipole is calculated by integrating the radial component of the Poynting vector over a closed spherical surface centred on emitter^3^. The collection efficiency is computed from the ratio of the power emitted in a specified angular range to the total power emitted over a closed surface surrounding the emitter. SPE is calculated by taking the ratio of the emitted power of a dipole in a nanodiamond (P_*E*_) in the cavity to the emitted power of the same nanodiamond in air (P_*Air*_) (SPE = P_*E*_/P_*Air*_). Here, the radial component of Poynting vector is integrated over a closed surface of same radius to find the emitted power in the above two cases. The SPE varies with respect to position of the dipole in the antenna. Thus, the enhancement and emission angle have been calculated at various dipole positions. A maximum enhancement of SPE = 20 is obtained without losing the high collection efficiency of more than 80% for ǁ, _┴_ (and 45°) dipole orientations.

**Figure 6:**
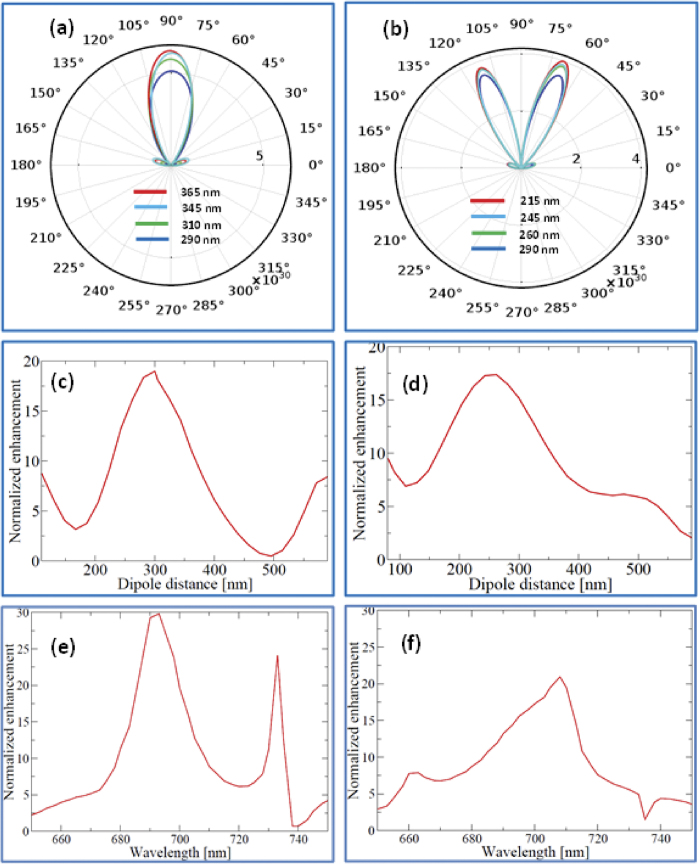
Polar far field radiation intensity patterns with respect to the position of dipole in the cavity to show that emission cone angle is insensitive over a range of distances (**a**) for ǁ dipole orientation (75 nm along *z* axis) (**b**) for _┴_ dipole orientation (85 nm along *z* axis) (**c**) Normalized cavity enhancement (SPE) with respect to vertical distance (*z* axis) from bottom of the cavity for ǁ dipole orientation (FWHM 144 nm) (**d**) for _┴_ dipole orientation(FWHM 195 nm). Normalized SPE for (**e**) ǁ dipole (FWHM 20 nm) (**f**) _┴_ dipole (FWHM 28 nm) with respect to wavelength range from 650 nm to 750 nm.

As a next step, we have varied the position of the nanodiamond vertically (*Z* axis as shown in the Fig. 4) to study the effect of any offset in the dipole position on the emission cone angle and the SPE. Figure 6(a,b) show polar plots of far field emission intensities with respect to emission angle for parallel and perpendicular dipole orientations over this range of 85 nm. The results show that the emission cone angle does not vary over the range. The SPE is calculated with respect to the position of the dipole in the vertical (*z* axis) as shown in Fig. 6(c,d). Along the *z* axis the variation in the peak emission enhancement is less with full width at half maximum (FWHM) 195 nm (_┴_) and 144 nm (ǁ) respectively. Over 80 nm offset in *x* axis, the reduction in the SPE is less than 10% and 20% for ǁ and _┴_ dipole orientations respectively.

## Discussion

These results show that the antenna is less sensitive to the position of the dipole over a range, which is well within any fabrication tolerances and this tolerance is enough to take into account random sizes of nanodiamonds with variable position of the dipole inside the nanodiamond, and also positioning of the nanodiamond via AFM or tungsten probe. To investigate the spectral response of the antenna, we scan the emission wavelength of the dipole in the cavity from 650 to 750 nm to determine the maximum enhancement. Figure 6(e,f) show the normalized enhancement in the emission rate obtained with respect to the wavelength for ǁ and _┴_ dipole orientations (normalized to the emission rate of an identical nanodiamond obtained in air). The distance of the dipole from the base of the cavity (*z* axis distance) is 315 nm and 215 nm for ǁ and _┴_ dipole respectively. Since NV centre is made of combination of two orthogonal dipoles, a suitable cavity parameter (position of dipole in the cavity, Z) can be obtained with maximum enhancement for both parallel and perpendicular dipoles from Fig. 6(c,d). The maximum enhancement obtained is ~30 with a FWHM of 28 nm (_┴_) and ~20 with a FWHM of 20 nm (ǁ). The geometry determined is suitable for any emitter, and the peak operating wavelength and the emission enhancement can be tuned by varying the waveguide parameters such as curvature, position of the dipole and refractive index of the waveguide used.

In summary, we have presented a novel micro-cavity waveguide antenna to enhance extraction of photons from NV center in nanodiamonds. The antenna manipulates the emission direction by making use of a combination of waveguide and a dielectric silver mirror. The antenna also enhances the spontaneous emission rate. Furthermore the geometry presented here is a unique solution to the difficulty in collecting photons from NV centres in nanodiamond which is associated with two orthogonal dipoles arranged in a plane perpendicular to the NV defect symmetry axis. Collection efficiencies of more than 80% are demonstrated irrespective of the dipole orientations using the antenna with an accompanying increase in the spontaneous emission rate by a factor of eighteen. We have also shown that the antenna geometry is robust against offsets in dipole positioning over a range of distances which falls well within any fabrication tolerances. The concave waveguide antenna will find potential applications in quantum cryptography, quantum computation, spectroscopy and metrology.

## Methods

The micro-cavity mirror antenna is computationally investigated using the finite element method (FEM) implemented in COMSOL MULTIPHYSICS 4.3b. A spherical simulation volume in 3D is used to find the emission characteristics such as the far field radiation profile, emission cone angle and enhancement in the spontaneous emission rate from an NV centre nanodiamond in the micro-concave waveguide antenna. In all cases, the nanodiamond is modelled as a point located at the centre of a sphere of radius 40 nm and refractive index 2.4. The emission wavelength is chosen at 700 nm vacuum wavelength (~spectral peak of the NV- luminescence in nano-diamonds at room temperature). The emission for parallel, perpendicular and 45 degrees dipole orientations are studied. The geometry consists of a concave cavity with radius of curvature 1200 nm (*R*_*s*_) on a semi- infinite semispherical silicon and covered with 300 nm thick silver layer (*T*_*Ag*_). The top surface of the silver coating has a curvature reduces of 900 nm (*R*_*Ag*_). The silver is covered with silicon nitride of thickness 615 nm and the nanodiamond is placed at 315 nm above bottom of the cavity inside the silicon nitride. Another semi-infinite half sphere of air is placed above the semi-infinite hemisphere silicon concave cavity. A scattering boundary condition is used around the sphere to truncate the simulation geometry. The radiated power intensity and far field radiation pattern is calculated using hollow spheres inside the geometry with the center of the sphere as the centre of the nanodiamond.

## Additional Information

**How to cite this article**: Rajasekharan, R. *et al.* Micro-concave waveguide antenna for high photon extraction from nitrogen vacancy centers in nanodiamond. *Sci. Rep.* **5**, 12013; doi: 10.1038/srep12013 (2015).

## Figures and Tables

**Table 1:** Collection efficiency from a dipole (ǁ and _┴_ orientation) in the micro-concave antenna for different NAs starting from 0.95 to 0.4 (parameters are same as for Fig. 5).

Numerical Aperture (NA)	Collection Efficiency (%)	
	_┴_	ǁ
0.95	92.0	90.0
0.9	89.0	86.4
0.8	87.0	83.8
0.7	86.5	83.5
0.6	82.2	83.0
0.5	69.1	79.1
0.4	51.2	72.3
